# Chickens selected for feather pecking can inhibit prepotent motor responses in a Go/No-Go task

**DOI:** 10.1038/s41598-020-63618-z

**Published:** 2020-04-16

**Authors:** Jennifer Heinsius, Nienke van Staaveren, Isabelle Young Kwon, Angeli Li, Joergen B. Kjaer, Alexandra Harlander-Matauschek

**Affiliations:** 1 Department of Animal Biosciences, University of Guelph, Guelph, Ontario Canada; 2 Friedrich-Loeffler-Institut Federal Research Institute for Animal Health, Institute of Animal Welfare and Animal Husbandry, Celle, Germany

**Keywords:** Motor control, Animal behaviour

## Abstract

Repetitive feather pecking (FP) where birds peck and pull out feathers of conspecifics could reflect motor impulsivity through a lack of behavioural inhibition. We assessed motor impulsivity in female chickens (n = 20) during a Go/No-Go task where birds had to peck (Go) or inhibit pecks (No-Go) appropriately to obtain a food reward, depending on visual cues in an operant chamber. Birds were selected to show divergent FP performance based on their genotype (high predisposition for FP or unselected control line) and phenotype (peckers or non-peckers). Genotype, phenotype, and its interaction did not affect the number of pre-cue responses, percentage of responses during No-Go cues (false alarms), or efficiency (number of rewards over number of responses). We present the first documentation of a Go/No-Go task to measure the ability of birds genetically and phenotypically selected for FP activity to inhibit a prepotent motor response. Results indicate that the repetitive motor action of FP does not reflect impulsivity and is not genetically linked to a lack of behavioural inhibition as measured in a Go/No-Go task.

## Introduction

Chickens use pecking as their main action for manipulation of objects, which include e.g., the handling of food, foraging, and exploring or moving items^1^. A similar pattern is seen in feather pecking (FP) – a behaviour where a bird reaches out to peck at the feather of conspecifics, which may or may not result in plucking of feathers and occasionally the consumption of feathers^2,3^. This damages the feather cover of conspecifics which in some cases can lead to cannibalism and ultimately death of the victim^3^. FP is a common behaviour in birds kept for egg-laying with a reported prevalence ranging from 15% to 95% on commercial farms^4–7^. This disruptive behaviour can be induced by adverse, stressful environments^8–10^ and is associated with neurobiological changes, such as alterations within the monoaminergic system^11^. This potential link to permanent neurobiological alterations, together with its apparent heritability^12,13^, makes FP difficult to treat and often irreversible^14^. Theoretically, neurobiological alterations can reduce behavioural control, and would explain functionless pecking by birds that engage in this behaviour during operant tasks^15–17^. Given the repetitive nature of FP, FP may be more akin to similarly described repetitive behaviour seen in human psychiatric disorders, such as attention-deficit hyperactivity disorder (ADHD)^18^.

Impulsivity is part of normal behaviour^19^. It is considered a favourable trait when decision making needs to be completed quickly, enabling the individual to seize fleeting opportunities, and when the outcomes of such decisions are positive. However, impulsivity can be detrimental when it is a persistent or a dominant trait (e.g., in people with psychological disorders such as ADHD)^20^. Impulsivity is a multifaceted construct that encompasses quick decision-making fuelled by a lack of forethought, decreased ability to pay attention, and decreased inhibitory control, among other traits^21^. Its complexity is evidenced by the multiple ways in which it is described in the literature and the different types of impulsivity that have been identified over the years^21^. One such subtype is motor impulsivity, which, on a cognitive level, is linked to an individual’s inability to suppress prepotent motor responses, otherwise known as response inhibition^22^. Responses are characterized as prepotent when an immediate positive or negative reinforcement is associated with it, and they are the dominant response to a given stimulus^23^. Motor impulsivity can be tested through Go/No-Go tasks which are applied in humans and in rodent models^20,24^. These tasks evaluate the ability of inhibitory control mechanisms to suppress rapid, conditioned motor responses (i.e., prepotent responses) allowing cognitive mechanisms to guide behaviour^20,25^. In this context, a Go cue would require the animal to perform a response, while a No-Go cue would require the animal to inhibit the same response. Highly impulsive animals are unable to accurately or fully execute action inhibitory control, and therefore, would be impaired in Go/No-Go task performance^20,26^.

To date, it is not clear whether motor impulsivity plays a role in FP behaviour in birds kept for egg laying. Our aim was to address this knowledge gap by investigating motor impulsivity in genetically- and phenotypically-selected FP hens. To this end, we compare motor impulsivity in a line of White Leghorns selected for high FP (HFP) to that of an unselected control line (CON) to determine the contribution of the respective genetic backgrounds^27^. While we hypothesize that the HFP line exhibits higher impulsivity in general, it has long been observed that FP behaviour does not occur in all individuals in a population with this genetic predisposition^28,29^. Consequently, the motor impulsivity between phenotypic peckers (P) and non-peckers (NP) within the HFP and CON lines was also evaluated. We predict that birds with a FP phenotype or genotype will exhibit higher motor impulsivity as shown by a lack of behavioural inhibition in a Go/No-Go task compared to their low pecking counterparts.

## Results

### Task acquisition

All hens used in the study were trained to peck upon receiving a Go cue and to abstain from pecking upon receiving a No-Go cue. This was achieved by shaping (phase 1–2) bird behaviour to the respective cues and encouraging the subjects to learn (phase 3–5) to perform the correct action with increasing speed (see also Methods, Fig. 3). Successfully performing either task awarded the bird a food reward and birds needed to earn at least 75% of the potential food rewards in two consecutive sessions to move through phases 3–5. In this task acquisition phase, pecking was transformed into a prepotent response where individual birds varied in the number of sessions they required to achieve this threshold (Table 1). On average, hens required 4.0 ± 0.47 sessions to move on to the next phase, with a minimum of 2 and maximum of 22 sessions. The average number of sessions required to learn the task was not impacted by the birds’ genotype (*F*_1,16_ = 0.13, *P* = 0.7203), phenotype (*F*_1,16_ = 2.44, *P* = 0.1381), or genotype-phenotype interaction (*F*_1,16_ = 0.02, *P* = 0.8997) (Table 1).

**Table 1:** The impact of phenotype and genotype on the learning ability of hens.

	Phenotype	Genotype	Number of Sessions
**Phenotype**			
P			3.2 ± 0.50
NP			4.3 ± 0.57
**Genotype**			
HFP			3.6 ± 0.50
CON			3.8 ± 0.58
**Interaction**			
	P	HFP	3.1 ± 0.64
		CON	3.2 ± 0.77
	NP	HFP	4.1 ± 0.78
		CON	4.6 ± 0.85

The effect of feather pecking phenotype (P: pecker n = 11, NP: non-pecker n = 9), genotype (HFP: high feather pecking line n = 10, CON: unselected control line n = 10) and their interaction on the learning ability of hens during training of a Go/No-Go task during task acquisition (phases 3-5) in a Go/No-Go task as measured by the average number of sessions (Least Square Means ± SE) required to advance to the next phase.

**Figure 1:**
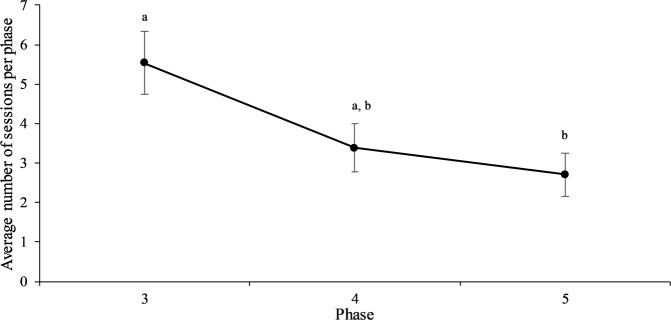
Average number of sessions (Least Square Means ± SE) required by hens to advance to the subsequent phase during acquisition of a Go/No-Go task (phase 3-5). Learning criterion to proceed to the next phase was set at 75% of rewards obtained over two consecutive sessions. Different letters indicate significant differences (P < 0.05).

All birds needed a significantly higher number of sessions (*F*_2,32_ = 4.77, *P* = 0.0154, Fig. 1) to pass the learning criterion in phase 3 compared to phase 4 (*t*_32_ = 2.12, *P* = 0.102) and phase 5 (*t*_32_ = 2.86, *P* = 0.0195), respectively. There was no difference in the number of sessions needed to proceed to the next phase in phase 4 and 5 (*t*_32_ = 0.81, *P* = 0.6974). Overall, this pattern demonstrates that the birds learned the task at hand over time, but that the FP behaviour did not impact task acquisition.

### Behavioural inhibition

To test motor impulsivity (phase 6) after birds had learned the task, the number of pre-cue responses and the percentage of false alarms were assessed. Pecking during the pre-cue period was considered an impulsive action as it indicates that the bird engaged their prepotent response in anticipation prior to receiving the appropriate stimulus. False alarms are defined as pecks in response to the No-Go cue, which indicates the inability of the animal to inhibit their prepotent response via response inhibition.

**Figure 2:**
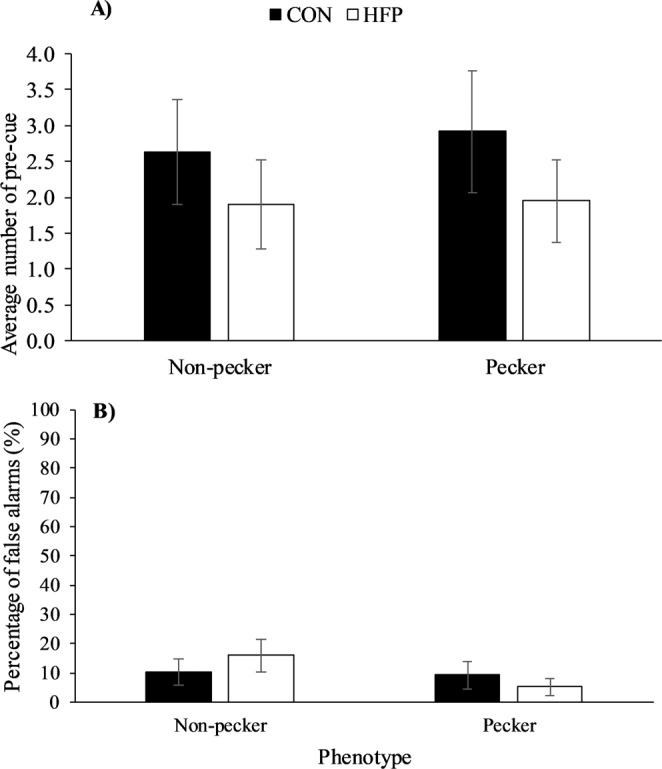
The average number of pre-cue responses (**A**) and false alarms (**B**) (Least Square Means ± SE) of hens with different feather pecking phenotypes (P: pecker n = 11, NP: non-pecker n = 9) and genotypes (HFP: high feather pecking line n = 10, CON: unselected control line n = 10) during a Go/No-Go task (phase 6).

We report that the birds’ phenotype, genotype, or the interactions thereof, did not affect the average number of pre-cue responses (Fig. 2A, genotype: *F*_1,16_ = 1.5, *P* = 0.2377, phenotype: *F*_1,16_ = 0.05, *P* = 0.8309, genotype x phenotype interaction: *F*_1,16_ = 0.02, *P* = 0.8985) or the average percentage of false alarms (Fig. 2B, genotype: *F*_1,16_ = 0.01, *P* = 0.9163, phenotype: *F*_1,16_ = 1.71, *P* = 0.2100, genotype x phenotype interaction: *F*_1,16_ = 1.19, *P* = 0.2924) when tested in phase 6. Additionally, phenotype, genotype, and their interaction did not influence the average number of correct pecks during Go cues (genotype: *F*_1,16_ = 2.09, *P* = 0.1675, phenotype: *F*_1,16_ = 0.35, *P* = 0.5604, genotype x phenotype interaction: *F*_1,16_ = 1.94, *P* = 0.1828) or correct withholds during No-Go cues (genotype: *F*_1,16_ = 0.01, *P* = 0.9163, phenotype: *F*_1,16_ = 1.71, *P* = 0.2100, genotype x phenotype interaction: *F*_1,16_ = 1.19, *P* = 0.2924). Finally, the response efficiency, defined as the number of rewards per number of responses, was not dependent on the birds’ genetic background, the FP behaviour or any potential interaction between these two factors (genotype: *F*_1,16_ = 0.02, *P* = 0.9038, phenotype: *F*_1,16_ = 0.25, *P* = 0.6209, genotype x phenotype interaction: *F*_1,16_ = 0.21, *P* = 0.6544).

## Discussion

We investigated the association between repetitive FP and motor impulsivity in birds that were selected for high FP behaviour (HFP birds) and those from a control line (CON birds). Prior to this study, a Go/No-Go task that can quantify impulsive behaviour had not been used in laying hens to assess the relationship between FP and motor impulsivity before. We hypothesized that FP is an impulse-driven behaviour that may be caused by neurobiological dysfunction with a genetic component^11^, and that it is similar to human psychiatric disorders that display repetitive behaviour (e.g., ADHD). This theory is supported by bird lines that are bred for high FP activity which is accompanied by high general locomotor activity^18^. We predicted that the genetic background that is associated with high FP behaviour will also contribute to impulsive behaviour involving motor actions in general. Consequently, birds that were genetically or phenotypically categorized as birds that perform FP would score differently on measures of impulsivity (higher number of pre-cue responses and false alarms, lower efficiency) in a Go/No-Go task relative to control birds. Against our predictions, we did not find an increase in motor impulsivity in domestic chickens that were genetically (HFP) and phenotypically (P) more inclined to feather peck as measured by the number of pre-cue responses, false alarms or the efficiency of each group to obtain the food reward.

The results of our study showed that HFP and CON birds were similarly able to inhibit pecking behaviour towards a visual cue in the operant chamber, suggesting that the genetic make-up of birds did not affect motor impulsivity. Interestingly, HFP birds exhibit higher general locomotor activity levels^18^, higher pecking activity at inanimate stimuli on a computer screen^30^, and at a pecking key^16^ than unselected CON lines. Theoretically, these repetitive behaviours could be associated with a lack of response inhibition. Alternatively, FP is thought to be a sustained response to the unnatural and stressful environments in which laying hens are kept^31^. It is, therefore, possible that FP is an inappropriate repetitive response in the absence of a stimulus or to an incorrect stimulus, also known as perseveration, which has close ties to impulsivity^32^. Kjaer *et al*.^33^ tested the frequency of recurrent perseveration in birds divergently selected for FP and a control line. Interestingly and contrary to their initial hypothesis, the authors reported that divergently selected birds were not associated with increased recurrent perseveration responses^33^. In fact, HFP birds showed a lower tendency for unnecessary repetitive responses than CON birds and a similar latency to repeat the same response in a two-choice guessing task as their CON counterparts^33^. It is noteworthy that while we recorded a lower number of pre-cue responses in HFP compared to CON birds, suggesting that HFP birds performed marginally better than CON birds, this observation was not statistically significant. It is possible that the relatively small sample size may hide significant effects. Nevertheless, the results reported are relatively similar across conditions to those reported by Kjaer *et al*.^33^. Given that the latter used a larger sample size, the observations presented in this study may be considered robust.

This is in contrast with findings on stereotypic behaviour in parrots where researchers found a positive association between stereotypic behaviour (including self-plucking of feathers) and recurrent perseveration^34^. The same study reported that rapid repetition of responses were observed in birds with a higher incidence of stereotypic behaviour^34^. As such, the present study and literature suggest that repetitive FP movements may not accompany motor impulsivity, or related cognitive problems and that they are not stereotypic in HFP birds.

Our results also suggest that phenotypic FP is not associated with motor impulsivity. Neither P nor NP birds differed in their responses during the Go/No-Go task showing the same amount of pre-cue responses, false alarms, and the same efficiency during the task (phase 6). Indeed, this shows that P birds were not impaired in task performance, as they were able to wait periods of unpredictable time (pre-cue period) without pecking a key, and they were able to suppress a prepotent motor response to the No-Go stimulus (false alarms, efficiency) similarly to NP birds. This mirrors the conclusions of a self-control study conducted in laying hens where birds anticipated future consequences by displaying high temporal cognitive abilities^35^. It is important to highlight that the present study was conducted solely in adult birds as FP behaviour is more mainly reported and observed in adult birds^7,14,36,37^. As such, there exists the possibility that P birds may indeed have impaired impulse control at a young age; however, they may develop coping strategies that diminish or mask this deficiency over time, thereby allowing P birds to perform Go/No-Go tasks similarly to their NP counterparts as adults. According to this train of thought, the lack of inhibition would be detectable in young birds, but not necessarily in adult birds. This hypothesis requires further study to be validated. It also noteworthy that P birds approached a tendency to learn the task at hand faster than the NP birds. Given this observation and the previous study by Kjaer *et al*.^33^, it is possible that a larger study cohort may unveil that P birds have better response inhibition capabilities than NP birds. Finally, FP may be assumed to be a goal/reward-oriented behaviour, where the goal is plucking and eating feathers. Consumption of feathers could potentially have beneficial effects on the gastrointestinal tract in birds^2,38,39^. Nevertheless, goal-directed behaviour in mammals is not associated with motor impulsivity^40^, which supports the observations within the current study.

While the data presented in this study do not support a correlation between FP with motor impulsivity, certain limitations of the study should be acknowledged. It is possible that the sensitivity of the Go/No-Go task procedure was not adequate to capture the genetic or phenotypic differences. For instance, although the procedure is well-established in mammals^26^, and settings were determined in pilot studies, it is possible that other pre-cue timings, cue light lengths, or food reward periods would have elicited genetic or phenotypic differences in the observed responses. Additionally, manipulation of the prepotent response by increasing the number of Go cues relative to the number of No-Go cues within a session could potentially negatively impact the No-Go performance.

As outlined earlier, the relatively small sample sizes used in this study may have impacted the results. To mitigate this, we employed an alternative strategy that uses population extremes relative to a larger random sample of birds, which is expected to yield the largest genotypic and phenotypic differences^27^. Consequently, we identified P and NP birds in the HFP and CON lines. This, in turn, is expected to yield valid inferences regarding the relative ranking of the different group^41^. Additionally, one can argue that selecting the highest peckers (P) in a population of HFP birds constitutes a continuation of selection^29^, as the selection of extreme phenotypes is a common breeding tool. It is also possible that the FP levels in the tested population were too low to make a significant association to motor impulsivity; however, it should be noted that FP levels are difficult to control and distinct FP groups may be formed and re-formed even after the birds are categorized into their FP groups, as the phenotypes may evolve over time^42^. Finally, all hens in the current study learned to successfully perform the required motor task, though there was variability between individual birds in the number of sessions required to reach the learning criterion. Homogeneity in task acquisition across phases 3–5 may be used as a sign of similar cognitive functioning between birds of different groups, regardless of FP status. In humans, some forms of intelligence can have a protective effect on self-regulatory behaviour^43^, however, whether certain cognitive abilities in hens impact motor impulsivity needs further investigation.

To our knowledge, this is the first study investigating a potential relationship between FP and motor impulsivity. Bird lines selectively bred for FP performed similarly to an unselected control line when tested for motor impulsivity using a Go/No-Go task. Similarly, we observed no difference between phenotypic peckers and non-peckers. This implies that birds that are identified as peckers by their genetic background or phenotypically were capable of inhibiting prepotent responses to the same extent as control birds or non-pecker birds, respectively. Our results suggest that FP is not associated with motor impulsivity as tested in a Go/No-Go task. Nevertheless, further investigations are required to determine the contribution of other types of impulsiveness to FP and other cognitive or non-cognitive abilities correlated with FP (e.g., motivation for FP) that can be used to differentiate peckers from non-peckers.

## Methods

This study was approved by the University of Guelph Animal Care Committee (Animal Utilization Protocol Number 3206). The study was carried out in accordance with relevant guidelines and regulations.

### Animals and housing

Birds with a high FP activity were part of a flock of 132 White Leghorn hens (64 weeks of age) that consisted of 84 high feather pecking (HFP) and 48 unselected control (CON) birds. Both lines originated from a selection experiment in which birds of the HFP line were identified based on the highest FP behaviour within that group^27^.

Birds were housed in 12 groups of 11 ± 1.5 birds (6 ± 0.9 HFP, 5 ± 1.1 CON) per pen under natural daylight and darkness for the duration of the experiment at the University of Guelph, Guelph, Canada. They were housed in identical enriched floor pens (118 L × 118 W × 365 H cm) containing two elevated perches (100 and 110 cm long) which were mounted at approx. 60 and 30 cm heights, wood shavings (5 cm depth) as a litter substrate, and equipped with one nest box, bell drinker, and feed trough. Commercial laying hen feed and water were provided ad libitum.

All birds were individually wing-tagged and identified by numbered soft silicone plates fastened to the backs of the birds using elastic straps around their wings for individual identification. One camera (Samsung SNO-5080R, IR, Samsung Techwin CO., Gyeongi-do, Korea) was mounted at the top of each pen to record the birds’ FP behaviour for 20 min/day on three consecutive days by a blinded observer. A FP event was characterized as forceful pecking, plucking and pulling on the feather cover of other birds. Although HFP birds showed a higher FP activity (1.4 ± 0.13 FP bouts) than CON birds (1.0 ± 0.16 FP bouts, F_1,102_ = 5.03, P = 0.0271), there was overlap among birds of the two lines. Therefore, we chose to compare a total of 20 birds that expressed extreme phenotypes in terms of FP behaviour^41^, where peckers (P) performed a minimum of 5 pecks/hour compared to non-peckers (NP) which performed less than 2.5 pecks/hour. This selection resulted in four distinct groups (HFP/P n = 6; HFP/NP n = 4; CON/P n = 5; CON/NP n = 5).

### Test equipment

A custom-made non-transparent polycarbonate operant conditioning chamber (60 L × 37 W × 60 H cm) was used to test impulsive responses (Med Associates, St. Albans, VT, USA). An LED house-light (yellow) was installed in the top of the chamber^17^. A pecking key was located 40 cm above the floor of the chamber and was programmed to illuminate either red or green. Additionally, it could be paired with a high frequency sound (3,035 Hz). Reinforcements were delivered via a feed trough (13.5 L × 4.5 H cm) located at the centre of the test panel, below the lighted pecking key and 25 cm above the floor. Access to the food reward was withheld and blocked off by a metal barrier manually until the pecking key was successfully pecked (Go cue) or not pecked (No-Go cue). The number of pecks and light cues delivered were automatically recorded through the Trans IV computer program (Med Associates, St. Albans, VT, USA). These recordings were validated through video recordings made of each session using a camera (JVC GC-PX100BU HD Everio) remotely connected via an iPad (Apple, Inc.) to allow the experimenter to observe each bird without visually distracting them.

### Testing protocol

The Go/No-Go testing protocol consisted of six phases and followed a similar procedure as used in mice and described by Wilhelm *et al*.^44^.

#### Task acquisition

**Figure 3:**
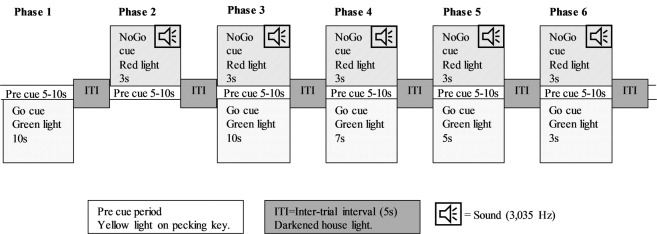
Description of the six different phases used throughout the Go/No-Go experiment.

Birds were individually acclimated to the operant conditioning chamber before testing by providing a luxury food reward (i.e., mixture of corn kernels, dead mealworms, and honey) within the chamber for five minutes once a day for 7 days. Learning to operate the pecking key was conducted through shaping and training (phase 1–5; Fig. 3). Once testing commenced, birds were not food-deprived and were tested five days per week. Each hen was only tested once per day for a total of 5 minutes.

The birds’ behaviour was shaped in response to Go and No-Go cues in phases 1 and 2 (Fig. 3). In phase 1, birds learned to peck the pecking key when its light (red or green) was illuminated in exchange for the food reward. Ten hens were trained to peck in response to a green light, and ten hens were trained to peck in response to a red light as the Go cue. Birds had to peck within 10 seconds after the Go cue was illuminated in order to terminate the lighted cue and receive 3 seconds of food reward access. In phase 2, birds were exposed to 3 seconds of the No-Go cues paired with a continuous 3.035 Hz tone, so that both light and sound stimuli were associated with the No-Go cue as described in Wilhelm *et al*.^44^. Birds had to withhold their pecking during this period in order to receive access to the food award. Key illumination was followed by a 5-second inter-trial interval (ITI), where the house light was switched off. This was followed by a variable pre-cue period (5-10 sec) where the house light was switched on. Pre-cue pecking was recorded and was neither rewarded or punished; however, pre-cue pecking during the final 3 seconds resulted in re-setting of the pre-cue period. Sessions ended when 5 minutes had passed. Birds were transferred to the next phase when they reached the learning criterion of accessing 75% of the potential food rewards in two consecutive sessions.

In phases 3, 4, and 5, Go and No-Go cues were interspersed throughout a session (approx. 8 Go-cues and 8 No-Go cues) in order to train hens and increase their response time, thereby stabilizing their responses to reflect their learning ability (Fig. 3). Each hen received an average of 8 Go cues and 8 No-Go cues per session. When no pecking occurred for 5 minutes, testing was ended. The No-Go cue duration was always 3 seconds, and ITI remained consistently 5 sec. The Go cue duration gradually reduced from 10 seconds in phase 3, to 7 seconds in phase 4, and 5 seconds in phase 5. The learning criterion to move to the next phase was the same as during phase 1 and 2 (at least 75% of rewards obtained in two consecutive sessions), however now 75% of food rewards was obtained by either pecking during Go cues or withholding pecking behaviour during No-Go cues.

### Behavioural inhibition

Once all birds had completed phase 3–5 and their pecking responses were stable, birds’ motor impulsivity was assessed in phase 6. In phase 6, Go and No-Go cues were similarly interspersed throughout a session, but the Go cue duration was reduced to 3 seconds, while the No-Go cue remained at 3 seconds with a 5 second ITI (Fig. 3). Hens received an average of 9 Go cues and 9 No-Go cues per session (min: 6, max: 13 for each cue).

Phase 1 and 2 were used to shape hen behaviour to associate the Go cue and No-Go cue with their assigned colour of the pecking key. A food reward was offered for correctly pecking or withholding from pecking in response to the appropriate cue. No-Go cues were paired with a high frequency sound. Each trial was preceded by a pre-cue period and the chamber was darkened in between each trial (inter-trial interval).

### Data analysis

All data were analysed using SAS Studio (SAS Inst. Inc., Cary, NC). The assumptions of normally distributed residuals and homogeneity of variance were examined graphically with the use of QQ plots. Statistical significance was considered at *P* < 0.05 and tendencies are reported at 0.05 ≤ *P* ≤ 0.1. Values are presented as LS means ± SE, unless stated otherwise.

Phase 1 and 2 were excluded as these were shaping phases before actual training started in phase 3 to 5. A mixed model ANOVA was used to investigate effects of phase (3, 4, 5), phenotype (NP, P), genotype (CON, HFP), and their interactions on the number of sessions needed to pass to the next phase. A random statement was included to account for hens as a repeated measurement.

To assess the ability of the birds to inhibit prepotent responses, the average number of pre-cue responses (pecks during the pre-cue period) and number of false alarms (incorrect responses during the No-Go cue) were calculated^44^. Additionally, the average number of correct pecks during the Go cue, and the average number of correct withholds of responses during the No-Go cue were determined. Furthermore, the average efficiency for the Go/No-Go task was defined as the average number of food rewards/average number of pecks and was used to assess the birds’ ability to peck or withhold pecking appropriately. Data were analysed using an ANOVA model accounting for the phenotype (NP, P), genotype (CON, HFP), and their interaction.

## Data Availability

The datasets generated during and/or analysed during the current study are available from the corresponding author on reasonable request.
